# Salvinorin A and *Salvia divinorum*: Toxicology, Pharmacological Profile, and Therapeutic Potential

**DOI:** 10.3390/ijms26125588

**Published:** 2025-06-11

**Authors:** Sara Calado, Bruno Pires, Luana M. Rosendo, Tiago Rosado, Eugenia Gallardo, Ana Paula Duarte

**Affiliations:** 1 RISE-Health, Departamento de Ciências Médicas, Faculdade de Ciências da Saúde, Universidade da Beira Interior, Av. Infante D. Henrique, 6200-506 Covilhã, Portugal; sara.calado@ubi.pt (S.C.); bruno.pinheiro.pires@ubi.pt (B.P.); may.rosendo@ubi.pt (L.M.R.); tiago.rosado@fcsaude.ubi.pt (T.R.); egallardo@fcsaude.ubi.pt (E.G.); 2 Laboratório de Fármaco-Toxicologia, UBIMedical, Universidade da Beira Interior, Estrada Municipal 506, 6200-284 Covilhã, Portugal; 3 Grupo de Investigação Sobre Problemas Relacionados Com Toxicofilias, Centro Académico Clínico das Beiras (CACB), Universidade da Beira Interior, Av. Infante D. Henrique, 6200-506 Covilhã, Portugal

**Keywords:** *Salvia divinorum*, salvinorin A, toxicity, therapeutic effects

## Abstract

*Salvia divinorum* is a psychoactive plant presenting a complex pharmacological profile, attracting significant scientific interest due to its potential therapeutic applications and associated health risks. This review provides a comprehensive analysis of the toxic and therapeutic effects of *S. divinorum*, evaluating its potential medical applications while highlighting the risks associated with its consumption. Additionally, the review examines the plant’s recreational use, global consumption trends, and legal status. By synthesising current research, this article aims to clarify the implications of *S. divinorum* use and inform future studies on its pharmacological potential and regulatory considerations.

## 1. Introduction

*Salvia divinorum* is a perennial member of the *Lamiaceae* (mint) family and it is native to the Sierra of Oaxaca, Mexico. It is also referred to as “diviner’s sage,” “magic mint,” “mystic sage,” “ska Maria,” and “ska Pastora”. The Mazatecs used this psychotropic herb to treat various ailments, including rheumatism, headaches, diarrhoea, and abdominal swelling, as well as for shamanic and divinatory purposes [1,2,3,4]. The plant was initially recognised as a distinct species of *salvia* in 1962 by Carl Epling and Carlos D. Játiva of the University of California [5].

In recent years, the potent hallucinogenic effects of *S. divinorum* have led to its use as a substance of abuse, particularly by young people in the United States and Europe. The consumption of this herb has increased rapidly, particularly among adolescents and young adults, due to its widespread availability both online and in smartshops, where its distribution remains widely unregulated in most countries [6]. In general, the hallucinogenic efficacy of *S. divinorum* is comparable to that of other classical hallucinogens such as lysergic acid diethylamide (LSD). However, although both substances may be equally potent, *S. divinorum* has shorter-lasting effects than LSD [7].

**Figure 1:**
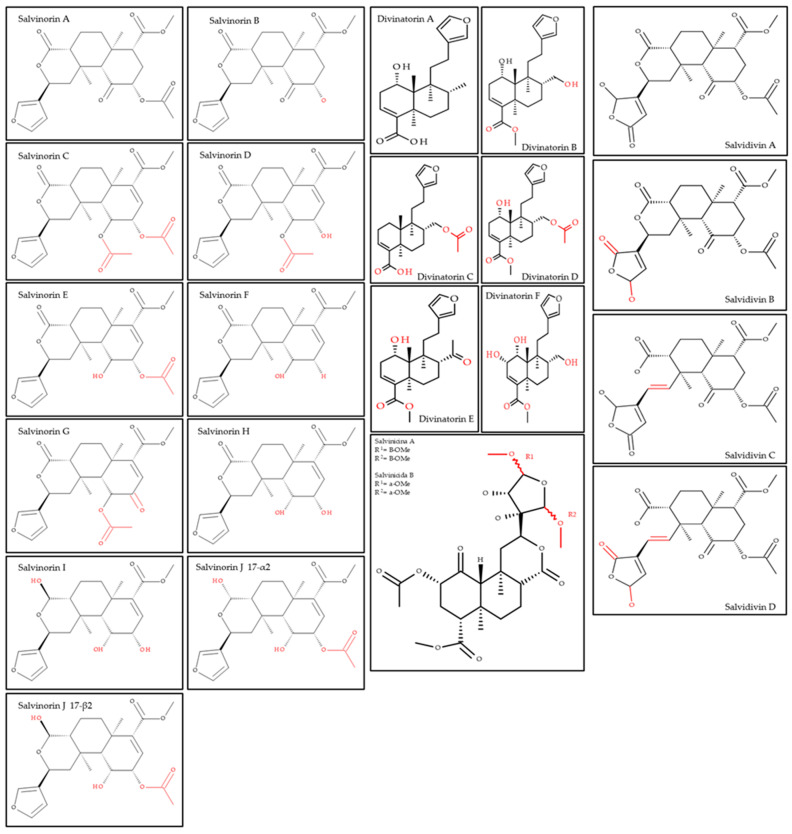
Chemical structures of the 22 diterpene-type compounds identified in *S. divinorum*.

At least 22 diterpene-type compounds have been identified in the phytochemistry of *S. divinorum*. Ten of them are classified as salvinorins (salvinorins A, B, C, D, E, F, G, H, I, and J—with salvinorin J occurring in two configurations: α and β), six as divinatorins (divinatorins A, B, C, D, E, and F), four as salvidivins (salvidivins A, B, C, and D), and two as salvinicins (salvinicins A and B) (Figure 1) [8,9].

The main bioactive compound of this plant is salvinorin A (SA), which belongs to the salvinorins group and is formed in glandular trichomes on the surface of the leaves of the plant. SA is responsible for the hallucinogenic effects [10,11,12]. SA has also been linked to the release and modification of monoaminergic neurotransmitters such as noradrenaline, dopamine, and serotonin. Clinical trials have demonstrated the beneficial effects of terpenes, with little to no adverse effects when used to treat pain [13].

SA is a neoclerodane diterpene that was first isolated and identified by Ortega et al. [14] and later by Valdes III et al. [15]. It is recognised as a selective and potent κ-opioid receptor agonist [14,15,16]. Classic hallucinogenic molecules, such as LSD, psilocin, DMT, and mescaline, activate serotonin-2A (5-HT_2_A) receptors, while SA is unique among psychoactive substances due to its selective agonism at the kappa-opioid receptor (KOR) [10,17,18].

The most common methods of administration for recreational users are smoking the leaves, crushing them to extract juice for oral consumption, or masticating and swallowing the whole leaves [19]. Two early reports indicate that the hallucinogenic effects of SA can last for up to an hour following conventional oral dosing [20]. Depending on the dosage, vaporisation or inhalation of SA results in an almost immediate experience of the full effects (30 s after consumption), which can quickly subside after intake [6,17,21,22,23]. Inhaling doses of 200–500 µg can result in effects lasting up to two hours [6]. In contrast, the effects of oral consumption take longer to manifest; they usually begin 5–10 min after ingestion and last longer, with the intensity of the effects increasing rapidly. This suggests that the oral mucosa acts as a release buffer, gradually releasing SA into the bloodstream [6,21].

**Figure 2:**
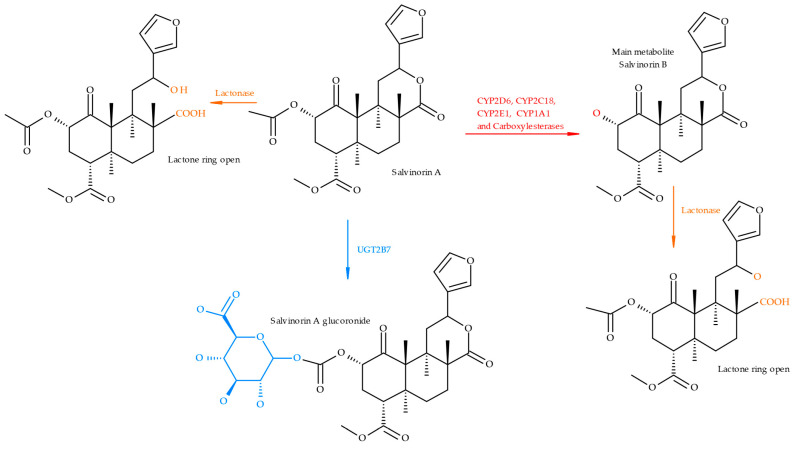
Metabolism of salvinorin A (SA).

SA exhibits numerous potential metabolic sites, including a lactone ring, a C_2_ acetyl group, and a C_4_ ester. The C_2_ acetate undergoes ester hydrolysis to produce salvinorin B, which has lower KOR activity, while the lactone group is known to be labile. The C4 ester has been proven to be enzymatically stable. Overall, it is believed that no active metabolites contribute to the effects of SA (Figure 2) [24].

Currently, few studies provide a complete in vivo toxicokinetic analysis of SA in humans, though some significant findings must be considered. Lipophilicity and molecular weight have long been thought to be the primary factors influencing drug permeation across the blood–brain barrier (BBB) [25]. SA is a highly lipophilic molecule, which may account for its rapid absorption in the central nervous system and its apparent ease of BBB penetration. In vitro studies suggest that SA is metabolised in humans by CYP450 enzymes (notably CYP2D6, CYP1A1, CYP2C18, and CYP2E1 isoforms), with glucuronidation likely serving as the drug’s primary metabolic pathway (Figure 2) [25]. Studies conducted in non-human primates have revealed interesting findings regarding SA toxicokinetics. According to Schmidt et al. [26], following intravenous administration of SA, effects appeared immediately and dissipated within 15 min, which is consistent with SA’s short duration of action. The total elimination half-life was 56.6 ± 24.8 min, with potential gender-specific variations in SA elimination, suggesting that its effects may differ between males and females.

This review aims to explore various aspects of *S. divinorum* with an emphasis on the toxic and therapeutic effects, addressing possible medical applications and health risks arising from its consumption. Furthermore, the plant’s recreational use, consumption statistics, and legal status will be discussed.

## 2. Toxicology and Pharmacokinetics

**Figure 3:**
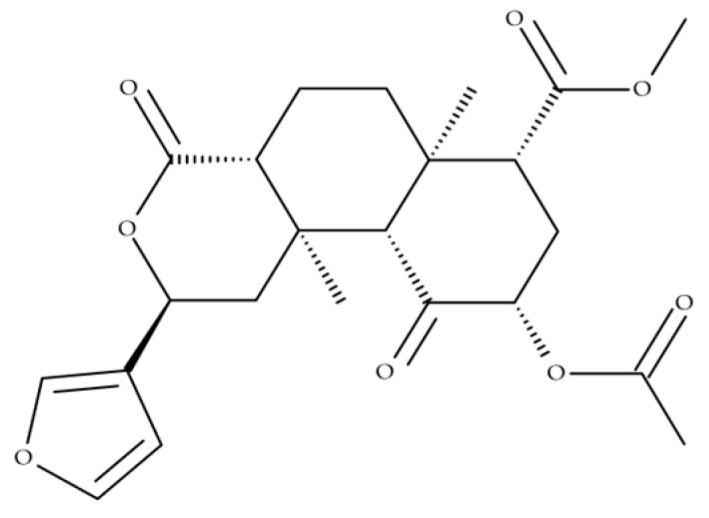
Chemical structure of salvinorin A (SA).

As previously stated, psychedelic drugs can cause a remarkable variety of changes in perception, emotion, and thought processes, with effects that are highly influenced by the user and the setting in which they are used [27]. SA (Figure 3) is a non-nitrogenous diterpenoid molecule that is the primary psychoactive ingredient in *S. divinorum*, although fewer active compounds have been isolated. SA, like ketamine, is regarded as a dissociative drug [6,20,21,22,23,27,28,29,30].

SA does not act on serotonin or dopamine receptors but targets KOR with high potency and selectivity. Notably, it is the first naturally occurring compound of its kind that lacks a basic nitrogen atom, diverging structurally from typical opioid ligands. Upon binding to KOR, SA initiates a cascade of intracellular events via Gi/o protein coupling, leading to the inhibition of adenylyl cyclase and a subsequent decrease in cyclic AMP (cAMP) levels. Additionally, the released βγ subunits modulate ion channels, inhibiting voltage-gated calcium channels and activating G protein-coupled inwardly rectifying potassium (GIRK) channels, ultimately promoting neuronal hyperpolarisation and reduced neurotransmitter release. Beyond these classical mechanisms, KOR activation by SA can also lead to extracellular signal-regulated kinase (ERK1/2) phosphorylation, which influences dopamine transporter function and may underlie some of the compound’s effects on mood, perception, and reward processing [10,17,18].

These intracellular pathways are critical not only for SA’s acute psychoactive effects, but also for its safety profile. By selectively engaging the KOR and avoiding interaction with mu-opioid (MOR) and serotonergic receptors, SA elicits atypical dissociative states without producing euphoria or cardiorespiratory depression. The downstream modulation of dopaminergic and glutamatergic systems may account for its hallucinogenic and anxiolytic properties, while also contributing to its low addictive potential [18,20]. Altogether, the molecular signalling pathways activated by SA, particularly its selective KOR engagement and associated intracellular cascades, are integral to understanding both its short-acting psychoactive profile and its pharmacological distinctiveness. These mechanisms not only distinguish SA from serotonergic hallucinogens but also support its emerging role as a scaffold for the development of functionally selective KOR ligands with potential clinical applications [18,31].

This lack of interaction with MORs and specific KOR modulation contributes to a lower risk of respiratory depression and other side effects often related to MOR modulation while also reducing the risk of addiction [21,28].

Additionally, SA does not affect 5-hydroxytryptamine receptor subtype 2A (5-HT_2A_R), the main molecular target of classical hallucinogenic drugs such as mescaline, psilocybin, and LSD [6,20,21,22,23,30]. It also exhibits a different behavioural profile from that of cannabinoids and has no affinity for the cannabinoid CB1 receptors in vitro [7,32]. Widely distributed throughout the central nervous system (CNS) and even in some peripheral tissues such as the intestines, lungs, kidneys, adrenals, spleen, stomach, testis, uterus, and ovaries, KORs are essential for controlling several physiological functions, including stress, pain, mood, reward, inflammation, and motor function [6,22].

This specific KOR affinity leads to a plethora of effects, with individuals usually reporting a disconnection from reality, visual and auditory hallucinations accompanied by object distortions, loss of contact with the body or depersonalisation, dizziness, unconsciousness, and motor discoordination [6,21,22,23,29]. Nevertheless, SA does not provide a sense of exhilaration or excitement, and it does not affect vital signs or cognitive impairments [21,23,33].

SA is primarily absorbed by the respiratory system and the oral mucosa, being quickly degraded in the gastrointestinal tract and metabolised in the liver and gallbladder, producing salvinorin B, its main inactive metabolite [6,20,21,22,23,32].

It is rapidly dispersed throughout the body, readily crossing the blood–brain barrier, where it accumulates and exerts its psychoactive effects before being quickly eliminated [6,17,21,22]. Its short psychoactive half-life (around 8 min) is closely associated with its pharmacokinetic characteristics and well correlated with its psychological effects [6,20,21,22,23].

Acute toxicity (1600 µg/kg) of SA in rats showed no impact on temperature, galvanic response, or cardiac conduction, while simultaneously being significantly less hazardous than morphine in humans (LD_50_ = 0.78 µg/mL for males and 0.98 µg/mL for females, respectively) [17]. Pure SA inhaled at concentrations as high as 12 mg has been shown to cause no harm or adverse effects in humans [17]. While most of the in vivo research indicates that SA and *S. divinorum* are relatively safe, in vitro toxicity in various cell lines (N 27, A 549, Caco 2, HepG2, Cos7, and Hek 293 cells) has shown dose- and time-dependent effects [6,17]. Additionally, Hep G2 and, to a lesser degree, Caco-2 cells have shown reduced susceptibility to *S. divinorum* and SA in contrast to N27, A549, COS-7, and HEK-293 cell lines [6,17]. Nevertheless, further knowledge regarding the complete scope of *S. divinorum* and SA toxicity in vivo, including both acute and chronic ingestion, is necessary to ensure its safe use.

## 3. Recreational Use and Legislation

The most popular methods of administration for recreational use include chewing and smoking the leaves [19,23,34]. Usually, a dose between 0.25 and 0.75 g of leaf material is smoked, producing effects within a minute and lasting up to 20 min [34]. A longer-lasting, mild to moderate effect is obtained by chewing the bitter leaves, typically 10–30 g of fresh leaves or 2–5 g of dried leaves. Drinking tea is uncommon since SA is easily broken down in the gastrointestinal tract [34].

Despite the potential dangers and laws enforced by each nation, the recreational use of this plant persists due to its extensive psychoactive properties. Recreational products, such as *S. divinorum* leaves and seeds, are easily accessible and can be purchased online and in physical stores that sell drug paraphernalia, such as head shops or smart shops, in countries or territories where their consumption is not prohibited [2,23]. These products are typically sold in packages containing leaves or another absorbent material impregnated with an extract of SA [2,23]. Although it is known by many street names, it is commonly marketed under the name “Purple Sticky™” in smoke shops [2,23,29,32].

The use of *S. divinorum* is strongly associated with alcohol, tobacco, cannabis, prescription and abused drugs, and other illegal substances, including LSD, ecstasy, heroin, phencyclidine, and cocaine [23]. Epidemiological evidence suggests that young adult white men with higher-income parents are more likely to use this substance. Additionally, a significant proportion of consumers report using *Salvia* primarily in their homes or apartments, highlighting the need for a secure setting to facilitate a smoother hallucinatory experience [23,29]. Individuals use *S. divinorum* for a variety of reasons, including curiosity and the desire to experiment with new substances. Others seek altered states of consciousness for spiritual experiences, self-discovery, or personal growth. Some consume it for meditation, relaxation, or enjoyment, while others use it recreationally to experience a high or an enhanced altered state. Additionally, some individuals turn to *S. divinorum* as a means of coping with psychological distress or to explore drug-induced states of altered consciousness [23].

Given that *S. divinorum* is often acquired through unregulated herbal products, special attention must be paid to the authentication of the plant material. Several morphologically similar *Salvia* species—particularly from the mint family—have been found to contain hepatotoxic compounds such as salviarin and rhyacophiline. Therefore, misidentification or contamination of commercial preparations may pose serious toxicological risks. Ensuring botanical accuracy is essential to avoid inadvertent exposure to harmful substances and to support the safe use of *S. divinorum*, whether for recreational or potential therapeutic purposes [4,35,36].

The scientific community remains divided concerning *S. divinorum*’s toxicity, potential for addiction, and medical applications, making its legal status a contentious issue [32]. In 2002, Australia became the first country to prohibit the sale and possession of *S. divinorum*, with several other nations following [32].

Currently, the legal status of *S. divinorum* varies by location and intended use. While it is not classified as a controlled substance at the federal level in the United States, the Drug Enforcement Administration (DEA) has identified it as a “drug of concern” due to its potential for misuse and abuse. It remains legal at the federal level but is restricted in eighteen states [37]. Some states impose no specific regulations, allowing legal use, possession, and sale, whereas others restrict their sales to individuals over 18 [37]. In Georgia, SA is classified as a dangerous drug, making its sale, distribution, or possession illegal, except when cultivated purely for decorative, landscaping, or aesthetic purposes [37]. Several European and Asian countries, including Belgium, Denmark, Italy, Latvia, Lithuania, Romania, Sweden, Australia, and Japan, have laws regulating *S. divinorum*. In Estonia, Finland, and Norway, it is allowed only for medicinal use, whereas in Croatia, Germany, Poland, and Spain, only the plant itself is regulated. In Canada, selling *Salvia* without authorisation is prohibited under the Natural Health Products Regulation [34].

## 4. Potential Therapeutic Uses

Historically, it has been employed in small doses to treat ailments such as diarrhoea, bloating, headaches, and rheumatism. The plant’s infusion or juice has also been used as a palliative remedy, helping to alleviate discomfort and promoting well-being [37].

SA is a potent and selective KOR agonist, which distinguishes it from classical psychedelics such as LSD and psilocybin, which primarily target the serotonin 2ª (5-HT_2A_) receptor. The unique pharmacological profile of SA has spurred interest in its potential therapeutic applications, ranging from pain relief to the treatment of mental health disorders. The following sections outline the therapeutic potentials described in the literature.

### 4.1. Pain Management

Chronic pain poses a significant medical challenge due to the side effects and dependency risks associated with conventional analgesics. Research has highlighted *S. divinorum* as a promising alternative for pain relief. SA’s interaction with KOR plays a crucial role in modulating pain perception, mood regulation, and motor control [37].

Studies have shown that SA produces antinociceptive effects without activating mu-opioid receptors, which are commonly associated with opioid addiction [37]. Pharmacological research demonstrates its dual agonist action on KOR and CB1 cannabinoid receptors, enhancing its analgesic potential [30,37,38].

In formalin-induced pain models, SA effectively reduced inflammatory pain and hyperalgesia. Aviello et al. [39] reported decreased paw oedema and nociceptive behaviour in mice, confirming its anti-inflammatory and pain-relieving properties [39]. Capasso et al. [40] further demonstrated that SA mitigated ileitis-induced hypermotility by modulating KOR and CB1 receptor interactions [40].

Recent studies suggest that SA may serve as a basis for developing short-term anaesthetics or non-addictive painkillers, given its fast onset and brief duration of action [37].

### 4.2. Mental Health Applications

#### Depression and Anxiety

The KOR agonism of SA has opened new avenues for treating mood disorders. Unlike serotonergic psychedelics, SA’s distinct mechanism offers an alternative approach to managing depression and anxiety.

A well-documented case study by Hanes [41] described a 26-year-old woman with chronic depression who achieved full remission after sublingual administration of *S. divinorum* leaves (0.5–0.75 g) three times per week for six months. Her progress, assessed using the Hamilton Depression Scale, revealed significant improvements.

Animal models have provided additional support. Rodents administered SA (0.001–1000 μg/kg, s.c.) exhibited reduced anxiety-like behaviour and increased exploration, indicating anxiolytic effects [19]. However, high doses (0.125–2.0 mg/kg) produced depressive-like symptoms and decreased locomotor activity, highlighting the importance of dose regulation [42].

Recent research also suggests that SA influences neuroplasticity by regulating brain-derived neurotrophic factor (BDNF) expression, which may underlie its antidepressant properties [43].

Despite promising preclinical data, clinical trials exploring *S. divinorum*’s therapeutic effects remain scarce. A Phase 1/2 trial registered on ClinicalTrials.gov investigated its impact on major depressive disorder but faced challenges with participant recruitment [44]. The open-label design and small sample size (only one participant at the time of review) limited the study’s conclusions.

### 4.3. Substance Use Disorders

SA has shown promising results in addressing substance use disorders by targeting the KOR-dopaminergic system. KOR activation reduces dopamine release in the nucleus accumbens, a brain region central to reward processing.

Preclinical studies by Glick et al. [45] and Appel and Kim-Appel [42] demonstrated that SA dose-dependently decreased cocaine and remifentanil self-administration in rhesus monkeys. This suggests that SA may diminish drug reward responses, offering a pharmacological route for treating addiction.

Rothman et al. [46] also found that SA disrupted drug-associated memory reconsolidation, reducing the risk of relapse. Furthermore, recent findings highlight its role in microglial polarisation—shifting from a pro-inflammatory M1 to an anti-inflammatory M2 phenotype—which could mitigate neuroinflammation linked to substance abuse [47].

### 4.4. Neuroprotection

Emerging research highlights SA’s neuroprotective potential, particularly in the context of cerebral ischaemia and brain injury. Studies have shown that SA can preserve vascular integrity and reduce inflammation.

In a middle cerebral artery occlusion (MCAO) mouse model, intranasal administration of SA (12.5–50 μg/kg) significantly reduced infarct volume, improved neurological outcomes, and increased IL-10 levels, supporting its anti-inflammatory action [48].

Furthermore, in models of hypoxic-ischemic injury in neonatal pigs, SA enhanced cerebral vasodilation and prevented apoptosis through nitric oxide synthase (NOS) and ATP-sensitive potassium channel (KATP channel) activation. This suggests it could play a therapeutic role in conditions involving cerebral vascular dysfunction [49].

### 4.5. Anti-Inflammatory and Antinociceptive Effects

SA’s anti-inflammatory properties extend to various models of inflammatory disease. Aviello et al. [39] found that doses of 0.5–2 mg/kg reduced carrageenan-induced paw oedema and formalin-induced inflammatory pain via KOR and CB1 receptor pathways. Additionally, SA inhibited leukotriene biosynthesis in activated rat peritoneal macrophages, reducing cell infiltration and vascular permeability. This suggests its potential for treating leukotriene-related conditions, such as asthma and cardiovascular diseases [50].

While *S. divinorum*’s hallucinogenic properties present certain challenges, its bioactive compound, SA, holds considerable therapeutic potential. From pain management and mood disorder treatments to neuroprotection and anti-inflammatory applications, its KOR agonism and interaction with cannabinoid receptors highlight a diverse pharmacological profile.

However, advancing its medical use hinges on rigorous clinical trials to balance therapeutic benefits with psychoactive risks. The ongoing exploration of SA and its analogues may pave the way for innovative, non-addictive treatments for pain, addiction, and mental health disorders.

### 4.6. Microdosing

Microdosing is the practice of consuming psychoactive substances in sub-perceptual dosages to acquire potential therapeutic advantages while avoiding full hallucinogenic or dissociative effects [51].

This approach has been extensively studied with classic psychedelics such as LSD and psilocybin, which have demonstrated promising results in improving mood, cognitive flexibility, and emotional resilience [51,52]. However, there has been little research into microdosing *S. divinorum*, particularly its primary active ingredient, SA.

Microdosing SA differs significantly from LSD and psilocybin, which act on 5-HT2A receptors and are commonly used for cognitive enhancement and mood regulation [51,52]. A study on microdosing found that while many users report benefits such as increased focus and creativity, a significant portion discontinued due to a lack of efficacy. Unlike LSD and psilocybin, SA targets the KOR receptor, producing short-lived effects that may require frequent dosing [51].

Ketamine, another KOR-interacting compound, provides prolonged antidepressant effects, whereas SA’s transient action presents challenges for sustained therapeutic use [51]. Similarly, ibogaine’s long-lasting influence on addiction pathways contrasts with SA’s rapid metabolism. Ayahuasca, a serotonergic psychedelic, and kratom, a partial opioid agonist, offer more sustained benefits and are more frequently used in microdosing practices [51].

While SA’s potential in mood regulation, pain management, and addiction treatment warrants further exploration, its distinct pharmacology and short duration necessitate targeted research to determine optimal dosing strategies and clinical viability.

Limited reports suggest that microdosing SA may influence mood, stress resilience, and pain perception [53,54]. Some users describe mild anxiolytic and antidepressant effects, while others report increased introspection and altered cognitive processing [54]. However, systematic studies are lacking, and concerns exist regarding KOR activation potentially inducing dysphoria. Unlike classical psychedelics, which promote sustained neuroplasticity, SA’s transient nature may require frequent administration to maintain any potential benefits.

Further research is needed to evaluate the efficacy and safety of SA microdosing, particularly its potential in mood disorders, pain management, and addiction treatment. Establishing optimal dosing strategies and understanding its long-term effects will be crucial for determining the viability in therapeutic applications.

## 5. Analysis of Salvinorin A in Biological Specimens

Given the significant role of *S. divinorum* and its primary active compound, SA, in both clinical and forensic toxicology, it is crucial to explore the methodologies available for their detection, the biological matrices that can be utilised, and the analytical techniques applicable to their identification. This section is based on a literature review conducted using the PubMed database with the search terms “*Salvia divinorum* and biological matrix” and “salvinorin A and biological matrix,” considering only human biological samples. The conventional matrices examined include whole blood, plasma, serum, and urine, while alternative specimens considered are oral fluid/saliva, hair, vitreous humour, sweat, and pericardial fluid. The selection process involved three independent reviewers, and only studies identified as relevant by at least two of them were incorporated into this analysis, covering publications from 2004 to 2025.

Despite the relevance of identifying these psychoactive substances, there are relatively few studies available, and the conclusions regarding the effects of their consumption remain limited. A comprehensive review by Margalho et al. [55] summarised the benefits and limitations of different biological samples and the analytical methods used for detection.

Table 1 presents an overview of the methodologies developed for detecting SA in biological samples. Among conventional matrices, plasma and urine are the most frequently used.

A noteworthy study by Barnes and Snow [56] investigated urine samples using both liquid–liquid extraction (LLE) and solid-phase microextraction (SPME) coupled with two-dimensional gas chromatography–time of flight mass spectrometry. The microextraction approach provided a detection limit (LOD) of 4 ng/mL and a quantification limit (LOQ) of 8 ng/mL, though it required a substantial sample volume (20 mL). Conversely, Moreno et al. [57] developed a method employing microextraction by packed sorbent with gas chromatography–tandem mass spectrometry using only 200 µL of urine, obtaining an LOD of 5 ng/mL and an LOQ of 20 ng/mL, with recoveries ranging from 72% to 80%.

Several studies have examined plasma and urine matrices using LC coupled to mass spectrometry. Schmidt and collaborators [58] utilised 500 µL of these samples, while Tidgewell et al. [59] developed a method for plasma analysis using SPE extraction, only using 250 µL. More recently, Caspers et al. [60] employed SPE extraction and LC-tandem mass spectrometry for 400 µL of plasma, achieving an LOQ of 0.05 ng/mL and recoveries between 93% and 114% [60].

Alternative biological samples have also been explored. Pichini et al. [61] assessed plasma, urine, saliva (1 mL), and sweat patches for SA detection. Using gas chromatography–mass spectrometry (GC-MS), they reported LODs of 5 ng/mL and LOQs of 15 ng/mL for saliva, along with an LOD of 0.003 µg/patch and an LOQ of 0.01 µg/patch for sweat, achieving recoveries above 77%. Later, the same research group [61] developed a method for detecting the compound in 25 mg of hair using ultra-high-pressure liquid chromatography–tandem mass spectrometry, with an LOD of 0.02 ng/mg and an LOQ of 0.05 ng/mg [62].

From a *postmortem* perspective, Margalho et al. [63] established a method for detecting SA in vitreous humour (100 µL) and pericardial fluid (250 µL), alongside blood and plasma samples. Using SPE extraction and GC-MS analysis, they reported excellent LOD and LOQ values of 5 ng/mL, with recoveries exceeding 79% [63].

Among the different approaches employed for the analysis of SA, SPE emerges as the most widely utilised technique, particularly due to its efficiency in selectively isolating analytes from complex biological matrices. Within SPE protocols, Oasis^®^ HLB (hydrophilic lipophilic balance) sorbents have demonstrated superior performance, likely attributed to their balanced retention of both polar and non-polar compounds, facilitating enhanced recovery rates and reproducibility. LLE remains a conventional alternative, particularly in studies involving urine and plasma. A critical assessment of LLE methodologies reveals that the most employed solvents include chloroform and binary solvent mixtures such as ethyl acetate–heptane and chloroform–isopropanol. The choice of solvent system is crucial, as it influences both extraction efficiency and the elimination of interfering compounds.

Despite the growing interest in miniaturised extraction techniques, their practical application in forensic and clinical toxicology remains relatively limited. However, some methodologies, particularly solid-phase microextraction (SPME) and microextraction by packed sorbent (MEPS), have gained recognition due to their potential for reducing solvent consumption and sample volume requirements. While these techniques align with green chemistry principles, their adoption has been constrained by factors such as lower sensitivity compared to conventional SPE and LLE methods.

Among the available instrumental techniques for detecting SA, liquid chromatography coupled to mass spectrometry (LC-MS and LC-MS/MS) is the most widely used approach. This preference is primarily due to its high sensitivity, specificity, and the fact that SA determination is often part of a multi-analyte method that enables the detection of other drugs of abuse, particularly those that are more thermally unstable, such as LSD. Although gas chromatography coupled to mass spectrometry (GC-MS and GC-MS/MS) is also used, especially in urine and hair analysis (Table 1), its application is comparatively less frequent than LC-MS-based methods.

In cases where the LOQ value was not specified, the lowest calibration curve point was considered.

## 6. General Discussion and Future Directions

Although mechanistic, toxicological, and therapeutic aspects of *S. divinorum* and SA have been discussed throughout this review, this final section aims to synthesise key insights and highlight future directions based on current gaps in the literature. SA remains a pharmacologically unique compound due to its potent and selective agonism of KOR, without significant interaction with serotonergic, dopaminergic, or cannabinoid systems [20,23,31]. This positions it outside the classical hallucinogen framework and underscores its relevance for studying alternative pathways of perception and consciousness.

The ability of SA to activate both G protein–dependent and β-arrestin–dependent pathways opens new opportunities for biased agonism research. Such functional selectivity could, in principle, allow for therapeutic benefits such as analgesia, anti-inflammatory, or antidepressant effects, while reducing the risk of adverse events like hallucinations or dysphoria. These pharmacological dimensions are still poorly explored in vivo, particularly in models that assess circuit-level changes or behavioural outcomes linked to specific signalling biases [16,17,31].

There is also a need for translational research bridging molecular pharmacology and clinical application. While preclinical studies suggest potential roles for SA in pain management, mood disorders, and substance use disorders, no placebo-controlled clinical trials have yet been conducted. Critical knowledge gaps remain regarding dose–response relationships, safety in humans, sex-related pharmacokinetic variability, and possible long-term neuroadaptations. Likewise, the impact of genetic variability in KOR expression or function warrants further investigation [19,21,26,33].

Future research should also explore the development of analogues or derivatives with improved pharmacokinetic profiles, such as extended half-life, enhanced bioavailability, and optimised receptor selectivity [11,18,22]. Finally, given the increasing non-medical use of *S. divinorum*, regulatory, ethical, and public health implications must be addressed alongside scientific developments [1,34].

## 7. Conclusions

*S. divinorum* and its active compound, SA, represent a pharmacologically distinct class of psychoactive agents due to their selective agonism of kappa opioid receptors (KOR). Unlike classical serotonergic hallucinogens, SA induces short-lasting dissociative effects through a unique mechanism of action. This review outlined current knowledge on its pharmacodynamics, toxicology, and potential therapeutic applications. While preclinical data indicate promise in areas such as pain, addiction, and mood disorders, translational and clinical research remains limited. Continued investigation is essential to clarify its medical value, address safety concerns, and explore its regulatory and public health implications.

## Figures and Tables

**Table 1:** Summary of methodologies for the detection of salvinorin A (SA) in biological samples.

Compounds	Sample/ Volume	Extraction Procedure	Analytical	LOD (ng/mL)	LOQ (ng/mL)	Recoveries (%)	Reference
Salvinorin A	Urine (20 mL)	LLE (chloroform). SPME (85 μm polyacrylate fibre)	GC × GC-ToFMS	200 for LLE; 4 for SPME	300 for LLE; 8 for SPME	n.s.	[56]
Salvinorin A	Urine (0.2 mL)	MEPS (C_18_)	GC-MS/MS (EI)	5	20	71.91–80.15	[57]
Salvinorin A Salvinorin B	Plasma and Urine (0.5 mL)	SPE (Oasis HLB (30 mg, 1 mL))	LC-MS (APCI)	2 (Salvinorin A in Plasma)	2 (Salvinorin A in Plasma) 50 (Salvinorin B in Plasma)	104.00–106.00 (Urine)	[58]
Salvinorin A	Plasma (0.25 mL)	SPE (Waters Oasis SPE cartridges)	LC-MS (APCI)	n.s.	1	n.s.	[59]
Salvinorin A	Plasma (0.4 mL)	SPE (Strata C_18_-E 55 micron, 70 Å)	LC-MS/MS (ESI)	n.s.	0.05	93.00–114.00	[60]
Salvinorin A	Plasma, Urine, Saliva, and Sweat (1 mL)	LLE (chloroform/isopropanol (9:1))	GC-MS (EI)	5 (Plasma, Urine and Saliva); 0.003 µg/patch (Sweat)	15 (Plasma, Urine and Saliva); 0.01 µg/patch (Sweat)	84.60 (Plasma); 93.70 (Urine); 84.20 (Saliva); 77.10 (Sweat)	[61]
Salvinorin A	Hair (25 mg)	LLE (Diethyl ether)	UHPLC-MS/MS (ESI)	0.02 ng/mg	0.05 ng/mg	79.60–97.40	[62]
Salvinorin A	Vitreous humour, Pericardial fluid, Blood, and Plasma (0.1– 0.25 mL)	SPE (Oasis^®^ HLB (3 mL, 60 mg))	GC-MS (EI)	5	5	79.60–100.60 (Vitreous humour); 93.40–100.20 (Pericardial fluid); 88.80–99.10 (Blood); 88.30–98.00 (Plasma)	[63]
Salvinorin A Salvinorin B	Urine and Plasma (1 mL)	LLE (DMSO/ PEG-400, (25:75, *v*/*v*)	LC-MS/MS (APCI)	n.s.	0.5	n.s.	[64]
Salvinorin A	Urine and Blood (1 mL)	SPE (Oasis^®^ HLB (3 mL, 60 mg))	LC-MS (ESI)	2.5	5	71.70	[65]

APCI: Atmospheric-pressure chemical ionisation; DMSO: dimethyl sulfoxide; EI: Electron impact; ESI: Electrospray ionisation; GC: Gas chromatography; GC × GC-ToFMS: Two-dimensional gas chromatography–time of flight mass spectrometry; LC: Liquid chromatography; LLE: Liquid–liquid extraction; LOD: Limit of detection; LOQ: Limit of quantification; MEPS: Microextraction by packed sorbent; MS: Mass spectrometry; MS-MS: Tandem mass spectrometry; n.s.: Not specified; PEG-400: polyethylene glycol 400; SPE: Solid-phase extraction; SPME: Solid-phase microextraction; UHPLC: Ultra-high-pressure liquid chromatography.
